# Differential expression of RBM5 and KRAS in pancreatic ductal adenocarcinoma and their association with clinicopathological features

**DOI:** 10.3892/ol.2012.1080

**Published:** 2012-12-17

**Authors:** JIE PENG, ALI KORD VALESHABAD, QINGFU LI, YUAN WANG

**Affiliations:** 1 Department of Gastroenterology, Xiangya Hospital, Central South University, Changsha 410008, Hunan, P.R. China;; 2 Department of Medicine and Division of Gastroenterology and Hepatology, The John Hopkins Medical Institutions, Baltimore, MD, USA

**Keywords:** pancreatic cancer, RNA binding motif 5, KRAS, clinicopathological features

## Abstract

RNA binding motif 5 (RBM5) is a tumor suppressor gene that regulates cell proliferation, differentiation and apoptosis through pre-mRNA splicing of related genes. This study aimed to detect RBM5 and KRAS expression in pancreatic ductal adenocarcinoma and their association with clinicopathological features. Detection of RBM5 and KRAS expression by quantitative reverse transcription-polymerase chain reaction (qRT-PCR) and western blotting was performed at mRNA and protein levels, respectively, in pancreatic cancer and non-tumor tissues. In addition, the association of RBM5 and KRAS expression with clinicopathological parameters and tumor recurrence was analyzed. The expression of RBM5 was significantly downregulated in pancreatic cancer tissues compared to peritumoral tissues at the mRNA and protein levels. Contrastingly, KRAS was significantly overexpressed in pancreatic cancerous tissues compared to peritumoral tissues. Analysis revealed that RBM5 expression was negatively correlated with KRAS expression in pancreatic cancer. Furthermore, reduced RBM5 expression has a close association with lymph node metastasis, distant metastasis, Union for International Cancer Control (UICC) stage and nerve and venous invasion, while overexpression of KRAS proteins was significantly correlated with tumor size, lymph node metastasis, UICC stage and nerve and venous invasion of pancreatic cancer. Significant RBM5 underexpression and KRAS overexpression were observed in pancreatic cancer compared to non-tumor tissues. There is a close association of differential RBM5 and KRAS with poor clinicopathological features, suggesting their potential roles in the progression and metastasis of pancreatic cancer.

## Introduction

Pancreatic ductal adenocarcinoma is one of the most lethal cancers with an overall 5-year survival rate of <5% (1,2). Only a small proportion of pancreatic cancers are eligible for radical resection and tumor recurrence is common following curative resection due to its extremely malignant potential and unusual resistance to chemotherapy and radiotherapy (3). Therefore, novel molecular biomarkers need to be developed and identified to improve early diagnosis as well as predictive prognosis.

The tumor suppressor gene, RNA binding motif 5 (RBM5) (also called Luca15 or H37), is one of the ∼45 genes located in the 370 kb tumor suppressor locus on chromosome 3p21.3. RBM5, through pre-mRNA splicing of multiple target genes, has been shown to function as a regulator of apoptosis. Its potential role in cell cycle arrest and apoptosis has been demonstrated in several malignancies, particularly non-small cell lung cancer (NSCLC) cells (4,5). KRAS, also known as guanosine triphosphatase (GTPase) KRAS, belongs to the RAS gene family which encodes for a small protein with a molecular weight of 21 kDa with GTPase activity. Mutation of a KRAS gene is an essential step in the development of a number of cancers, including pancreatic cancer. Overexpression of KRAS recruits and activates proteins necessary for the propagation of growth factors and other molecular signals, including c-RAF and phosphoinositide 3 (PI3)-kinase, as well as inactivation of p53 and DPC4/Smad4, which are involved in numerous signal transduction pathways (6–9). Previously, a reverse correlation between RBM5 and KRAS was reported in lung cancer tissues (10). In the present study, we investigated the expression of RBM5 and KRAS at mRNA and protein levels and their associations with clinicopathological features in pancreatic ductal adenocarcinoma.

## Materials and methods

### Patients and sample preparation

In this study, we collected 45 cases of surgically resected pancreatic ductal adenocarcinoma samples and adjacent non-tumor tissues from July 2005 to June 2010. Following surgical removal, all samples were immediately snap-frozen in liquid nitrogen and stored at −80°C until total RNA was extracted. Clinical data including age, gender, tumor pathological characteristics and tumor stage were also collected based on patient medical records. Tumors were staged according to the tumor node metastasis classification (11). All tumor diagnosis was confirmed by a pathologist using standard diagnostic criteria on the pathological sections.

The study was approved by the Medical Ethics Committee of Central South University, Xiangya hospital, Changsha, China.Written informed patient consent was obtained from the patient’s family.

### Reverse transcription-polymerase chain reaction (RT-PCR)

The expression levels of RBM5 and epidermal growth factor receptor (EGFR) mRNA were determined using a quantitative RT-PCR technique (12). Briefly, total RNA was isolated from samples using the TRIzol reagent (Invitrogen, NY, USA) according to the manufacturer’s instructions. Quantitative real-time PCR was performed on an ABI 7300 real-time PCR system (Applied Biosystems, CA, USA) using SYBR-Green mix (Applied Biosystems). Relative gene expression was calculated using the ΔΔCt method, following the manufacturer’s instructions. All reactions were carried out in triplicate. The primer sequences were: glyceraldehyde 3-phosphate dehydrogenase (GAPDH), 5′-GGTGATGCTGGTGCTGA GTATGT-3′ and 5′-AAGAATGGGAGTTGCTGTTGAA GTC-3′; RBM5, 5′-CAGCGCATATGGTTTGTCGG-3′ and 3′-TGCCCTTAAAGAGACAGGCG-5′; KRAS, 5′-ACT GGGGAGGGCTTTCTTTG-3′ and 5′-GGCATCATC AACACCCTGTCT-3′.

### Detection of RBM5 and KRAS protein levels by western blot analysis

Total cellular proteins from pancreatic tumor and peritumoral tissues were extracted according to the protocol and protein concentrations were determined using the Bradford method (Bio-Rad, Hercules, CA, USA). An equal amount (50 *μ*g) of proteins were separated by sodium dodecyl sulphate-polyacrylamide gel electrophoresis (SDS-PAGE) and subsequently transferred onto a polyvinylidene fluoride (PVDF) membrane (Millipore, Bedford, MA, USA). After washing with tris-buffered saline (TTBS), membranes were incubated with the primary antibodies including anti-human RBM5 and KRAS antibodies (Abcam, MA, USA) as well as an anti-β-actin antibody (Santa Cruz Biotechnology Inc., Santa Cruz, CA, USA) as a control, overnight at 40°C. Following this, incubation with the secondary antibody, immunoglobulin G-horse radish peroxidase (IgG-HRP; Abcam) was performed at room temperature for 45 min. Finally, the results were visualized with enhanced chemiluminescence (ECL) detection reagent and quantitated by densitometry using the ImageQuant image analysis system (Molecular Dynamics, Sunnyvale, CA, USA). A 50^th^ percentile cutoff point was used to define over- or underexpression in pancreatic ductal adenocarcinomas.

### Statistical analysis

A Wilcoxon matched pair test was used to compare the mRNA and protein expression of RBM5 and KRAS between pancreatic tumor and peritumoral tissues. Correlations between RBM5 and KRAS expression were tested by the Spearman’s rank test. The associations of RBM5 and KRAS expression with the clinicopathological features were examined with the Chi-square test or Fisher’s exact probability test. Tumor recurrence rates were calculated using the Kaplan-Meier method and compared by means of the log-rank test. Factors with tumor recurrence rate in the univariate models were further evaluated in a multivariate Cox regression model. All statistical analyses were performed using SPSS software, version 19.0. (SPSS, Chicago, IL, USA). P≤0.05 was considered to indicate a statistically significant difference.

## Results

### Expression of RBM5 decreases in pancreatic ductal adenocarcinoma

**Figure 1:**
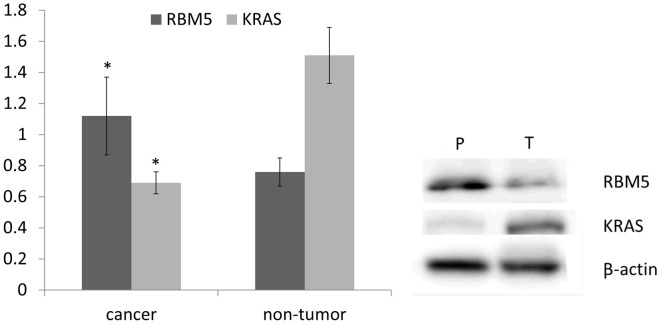
Relative expression of RBM5 and KRAS at mRNA and protein levels in pancreatic ductal adenocarcinomas and peritumoral tissues. The left panel reveals that cancerous tissues had a significantly decreased expression of RBM5 mRNA and increased expression of KRAS mRNA, compared with peritumoral tissues (^*^P<0.05). The right panel shows the western blot analysis results for RBM5 and KRAS protein expression in pancreatic tumor and peritumoral tissues (P, peritumor tissue; T, tumor tissue). RBM5, RNA binding motif 5.

To assess the expression of RBM5 mRNA and protein in pancreatic ductal adenocarcinoma, we performed quantitative RT-PCR and western blot analysis on 45 pairs of cancerous vs. peritumoral tissues. Compared to the peritumoral tissues, RBM5 was downregulated in pancreatic cancers (P<0.05; Fig. 1). Consistent with the mRNA expression level, the protein level of RBM5 detected by western blot analysis, was significantly lower in cancerous tissues than the peritumoral counterparts. Our results revealed that the expression of RBM5 mRNA and protein is significantly decreased in pancreatic ductal adenocarcinoma in comparison to peritumoral tissues (P<0.05; Fig. 1).

### KRAS expression increases in pancreatic ductal adenocarcinoma

Quantitative RT-PCR and western blot analysis were also performed to detect the expression of KRAS mRNA and protein in 45 pairs of pancreatic cancer vs. peritumoral tissues. The relative KRAS mRNA expression was significantly higher in tumor tissues than that in non-tumor tissues. Similarly, KRAS protein expression detected by western blot analysis demonstrated a marked increase in tumor tissues compared to peritumoral tissues. Our results revealed that KRAS is significantly overexpressed in pancreatic ductal adenocarcinoma in comparison to the non-tumor counterpart (Fig. 1).

### RBM5 and KRAS protein expression correlation in pancreatic ductal adenocarcinoma

By Spearman’s rank test, we analyzed the correlation between protein expression of RBM5 and KRAS in 45 pancreatic ductal adenocarcinomas. The results indicate that the expression of the RBM5 protein is reversely correlated with the expression of the KRAS protein in pancreatic ductal adenocarcinomas (R=−0.892, P<0.01).

### Association of RBM5 and KRAS expression with clinical features of pancreatic ductal adenocarcinoma

The associations of RBM5 and KRAS expression with clinicopathological features of pancreatic ductal cancers are summarized in Table I. As shown in the table, underexpression of RBM5 in pancreatic cancers was found to be significantly associated with lymph node metastasis, distant metastasis, Union for International Cancer Control (UICC) stage and nerve and venous invasion (P<0.05). No significant differences were identified between the RBM5 expression and the clinicopathological parameters with respect to age, gender, tumor size or cell differentiation (P>0.05). In contrast to RBM5, overexpression of KRAS was significantly correlated with tumor size, lymph node metastasis, UICC stage and nerve invasion (P<0.05). No significant difference was found between age, gender, distant metastasis, cell differentiation and venous invasion (P>0.05).

### Tumor recurrence and expression levels of RBM5 and KRAS

**Figure 2:**
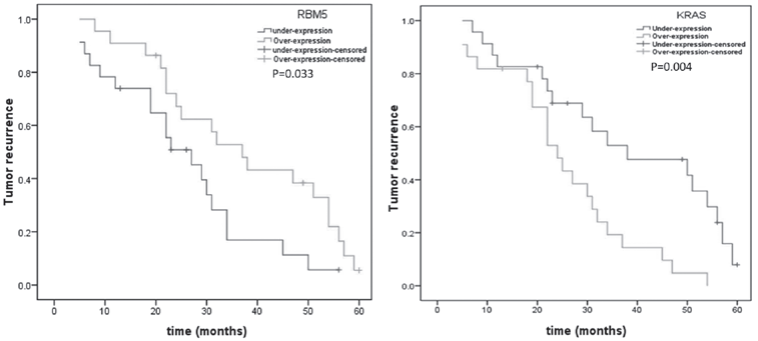
Postoperative recurrences for pancreatic cancer according to differentially expressed RBM5 and KRAS was analyzed by the Kaplan-Meier method. Significant associations of higher tumor recurrence rate with RMB5 underexpression and KRAS overexpression were found, respectively. RBM5, RNA binding motif 5.

Tumor recurrence, analyzed by the Kaplan-Meier method, suggested that underexpression of RBM5 had a greater incidence of recurrence than overexpression (P=0.033; Fig. 2). Additionally, a significantly increased tumor recurrence rate was related to overexpression of KRAS in pancreatic cancers in comparison to that with underexpression (P=0.004; Fig. 2). Further multivariate analyses using Cox’s proportional hazards model indicated that lymph node metastasis, tumor size (>4 cm), advanced UICC stage and nerve invasion have predictive value with regard to tumor recurrence of pancreatic cancer following radical resection (Table II). However, RBM5 and KRAS expression was not an independent factor associated with a higher rate of tumor recurrence.

## Discussion

In previous years, numerous molecular markers have been identified that are not only implicated in the occurrence and developmental progress of pancreatic ductal adenocarcinoma but also act as postoperative indictors for prognosis. Studies have shown a reduced expression of RBM5 in several cancers, including breast cancer, vestibular schwannoma and human non-small cell lung cancer (10,13,14). To our knowledge, this is the first study to investigate RBM5 expression at an RNA and protein level in pancreatic ductal adenocarcinoma.

In the present study, significantly reduced RBM5 expression was observed in pancreatic cancer tissues compared to peritumoral tissues. Additionally, underexpression of RBM5 had a close association with unfavorable clinicopathological features, including lymph node metastasis, distant metastasis, advanced UICC stage and presence of nerve and venous invasion, suggesting its potential role in tumor invasion and progression. Several studies have showed that RBM5 is differentially expressed in human cancers and is involved in tumorigenesis (15–18). Oh *et al*(19) found that the tumor suppressor RBM5/H37 alters the expression of genes involved in metastasis, suggesting that a loss of RBM5 expression may increase the metastatic potential of tumors. Our results, which demonstrated a significant association between reduced RBM5 expression and metastasis-related clinicopathological features support the above study. However, diverse effects of RBM5 have been revealed in human cancers. A previous study revealed that RBM6-RBM5 transcription-induced chimerism leads to tumor-related increased transcriptional activity of the RBM6 gene and has a close association with breast tumor size, suggesting that it may be a potential tumor differentiation marker (20). Taken together, it is clear that the role of RBM5 in pancreatic cancer is still not fully understood.

Oncogenic activation of the KRAS gene occurs in >90% of pancreatic ductal carcinoma and malignant progression from pancreatic intraepithelial carcinoma to a more aggressive form of pancreatic cancer is accompanied by the early acquisition of KRAS oncogene activation (21,22). The high frequency of KRAS mutations in pancreatic cancers has been proposed as a diagnostic tumor marker, as well as a prognostic indicator. Consistent with other studies, our results revealed that pancreatic cancer tissues are presented with elevated KRAS expression compared to non-tumor tissues (23–25). Moreover, our results also demonstrated a close correlation between KRAS overexpression and unfavorable clinicopathological factors, including larger tumor size, lymph node metastasis, advanced UICC stage and the presence of nerve invasion. However, taking into account the contrasting results regarding the prognostic utility of KRAS mutations, KRAS currently cannot be recommended for clinical application to determine prognosis in patients with pancreatic adenocarcinoma cancer (26–28). Further studies are required to understand the clinical implication and mechanism of KRAS in pancreatic cancer.

The correlation between RBM5, EGFR and KRAS has been demonstrated in several publications (4,13,29). RBM5 expression is altered as a result of changes in KRAS and the EGFR dimerization partner, human epidermal growth factor receptor 2 (HER2). A previous study demonstrated a reverse correlation between RBM5 expression and EGFR and KRAS expression in NSCLCs (10). However, another study indicated that reduced EGFR expression has no correlation with any change in RBM5 expression at either the RNA or protein level (30). Our results revealed that the expression of RBM5 and KRAS is negatively correlated in pancreatic cancer. Additionally, underexpression of RBM5 and overexpression of KRAS in pancreatic cancer has a close association with metastasis and invasion-related clinicopathological features, suggesting their collaboration in prompting tumor ability of invasion and metastasis.

Cancer recurrence is a major concern in patients with pancreatic ductal adenocarcinoma following radical resection. The differentially expressed RBM5 and KRAS in pancreatic cancer and their significant associations with postoperative recurrence of pancreatic cancer suggests their potential use as predictors of clinical implication. However, neither low RBM5 expression nor high KRAS expression were proved to be an independent factor associated with higher recurrence rate when corrected with age, gender, tumor size, lymphatic involvement, distant metastasis, cell differentiation, UICC stage and nerve and venous invasion. Our results are in accordance with the majority of studies, which suggest that KRAS mutations have no significant association with prognostic survival in pancreatic cancer patients (31–33). However, a number of conventional clinico-pathological parameters in our study, including lymph node metastasis, tumor size (>4 cm), advanced UICC stage and nerve invasion, are implicated as independent prognostic indicators for pancreatic cancer.

Collectively, expression of RBM5 and KRAS in pancreatic ductal adenocarcinomas is significantly decreased and increased, respectively, compared to non-tumor tissues. Furthermore, we revealed that poor clinicopathological features and high tumor recurrence rate are significantly associated with a low expression of RBM5 and high expression of KRAS. Further research is required to determine the role of RBM5 in metastasis and invasion of pancreatic cancer.

## Figures and Tables

**Table I:** Correlations between RBM5 and KRAS expression and the clinicopathological features of pancreatic cancer.

		RBM5 expression			KRAS expression		
Clinicopathological features	n	Under	Over	P-value	Under	Over	P-value
Age (years)							
<65	25	14	11	0.947	13	12	0.8940
≥65	20	11	9		10	10	
Gender							
Male	28	14	14	0.336	13	15	0.4200
Female	17	11	6		10	7	
Tumor size							
≤2 cm	16	11	5	0.348	13	3	0.0110
2–4 cm	17	9	8		6	11	
>4 cm	12	5	7		4	8	
Lymph node metastasis							
Absent	13	4	9	0.033	3	10	0.0165
Present	32	21	11		20	12	
Distant metastasis							
Absent	40	20	20	0.034	21	19	0.5980
Present	5	5	0		2	3	
Cell differentiation							
Good	18	11	7	0.179	12	6	0.0540
Moderate	13	9	4		5	8	
Poor	14	5	9		6	8	
UICC stage							
I or II	29	12	17	0.010	18	11	0.0480
III or IV	16	13	3		5	11	
Nerve invasion							
Absent	26	10	16	0.007	18	8	0.0044
Present	19	15	4		5	14	
Venous invasion							
Absent	33	15	18	0.024	15	18	0.2080
Present	12	10	2		8	4	

RBM5, RNA binding motif 5; UICC, Union for International Cancer Control.

**Table II:** Multivariate analysis of tumor recurrence for pancreatic cancer following radical resection.

Variable	HR	95% CI	P-value
Tumor size (≤4 cm vs. >4 cm)	4.510	1.1–11.34	0.034
Lymph node metastasis (absent vs. present)	4.030	1.17–13.88	0.027
UICC stage (I and II vs. III and IV)	4.050	1.03–9.54	0.044
Nerve invasion (absent vs. present)	5.482	1.14–26.3	0.033

HR, hazard ratio; CI, confidence interval; UICC, Union for International Cancer Control.
